# The Method of Evaluation of Radio Altimeter Methodological Error in Laboratory Environment

**DOI:** 10.3390/s22145394

**Published:** 2022-07-19

**Authors:** Pavol Kurdel, Marek Češkovič, Natália Gecejová, Ján Labun, Ján Gamec

**Affiliations:** 1 Faculty of Aeronautics, Technical University of Košice, Rampová 7, 041 21 Kosice, Slovakia; pavol.kurdel@tuke.sk (P.K.); marek.ceskovic@tuke.sk (M.Č.); 2 Faculty of Electrical Engineering and Informatics, Technical University of Košice, Letná 9, 042 00 Kosice, Slovakia; jan.labun@tuke.sk (J.L.); jan.gamec@tuke.sk (J.G.)

**Keywords:** depolarisation reflection panel, height impulse, methodological error, radio altimeter, safety

## Abstract

The presented article is focused on the evaluation of aviation radio altimeter (*ALT*) methodological error in order to increase air traffic safety. It briefly explains the background of methodological error at the theoretical level and offers practical conclusions to understand the issue. A radio altimeter provides information on an aircraft or helicopter’s instantaneous (radar) altitude or UAV to the pilot and another assistance system, such as an autopilot or an anticollision system. The height measurement of the most common used *ALTs* is realized with an accuracy of from ±0.30 m to ±0.75 m. This error rate corresponds to and is caused by the radio altimeter’s methodological error (Δ*H*). The *ALT* operating parameters are defined by carrier frequency, modulation frequency, and frequency lift. The methodological error of *ALT* can be obtained in three ways—calculated on a theoretical level, simulated in a suitable simulation environment, or evaluated in laboratory conditions. The ambiguity of *ALT* methodological error measurement causes bias in its presentation. This often leads to an incorrect determination of measurement inaccuracy (too optimistic statement of error value). The article’s primary goal is to present a new method for determining the value of the methodological error and its effect on the resulting error of measurement of the radio altitude (radar altitude). It presents a new experimental laboratory method for measuring Δ*H* and the resulting accuracy of height measurement with a radio altimeter. Thanks to this method, it can be verified that the information obtained by measuring the height above the ground corresponds to the standard specified by the manufacturer.

## 1. Introduction

**Figure 1:**
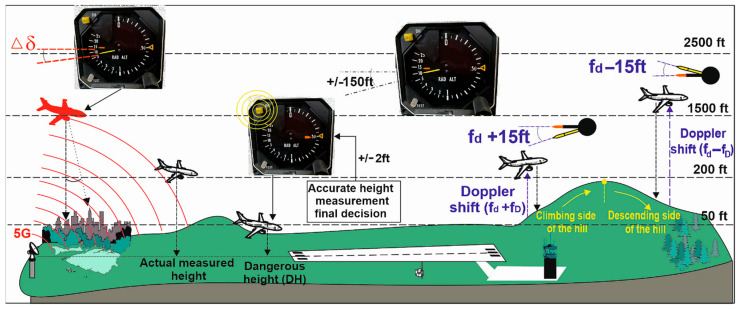
Visualization of various influences on height measurement accuracy by radio altimeter ALT.

One of the most critical parameters in terms of flight safety is the knowledge of the actual (radio) altitude of the aircraft above the surrounding terrain. This information is obtained from radio altimeters (*ALT*), which have been used in aviation since 1938 [1,2]. The principle of radio wave reflection from the earth’s surface enabled very accurate altitude measurements and much higher accuracy than measuring altitude with barometric altimeters or *GNSS* (geometric altitude vs. real altitude). Information about the radio altitude of the vehicle is critical not only for pilots in the approach and take-off phase in all meteorological conditions (Figure 1) but also for several avionics systems such as automatic flight control systems (Automatic Flight Guidance and Control Systems, Stick pusher/shaker, Flight Director, Thrust reverse, Autothrottle, Flight Controls, Envelope Protection Systems), anticollision systems (Enhanced Ground Proximity Warning Systems—EGPWS, Traffic Collision Avoidance Systems—*TCAS*, Windshear detection systems, Tail strike prevention system), and assistance aircraft systems (Primary Flight Display of height above ground, Take-off guidance systems, Engine and wing anti-ice systems) [2,3]. A radio altimeter (*ALT*) failure or malfunction can result in a disaster [3,4,5].

Current aviation radio altimeters (*ALT*) most often operate with frequency-modulated continuous-wave (*FMCW*) frequency modulation with an operating frequency in the 4.2–4.4 GHz band [1,2,3]. The principle of operation of *FMCW* radio altimeters has been used without significant changes for more than 50 years. The modernization of *FMCW ALT* consisted mainly of the addition of microprocessor technology and signal filtering, but the basic principle remained unchanged [1,2]. We can state that due to the long-term reliability and trouble-free operation of *ALT*, no significant attention was paid to these devices. The change did not occur indirectly until the last decade with the introduction of various environmental measures to reduce fossil fuel consumption. One way to save fuel is to reduce the weight of aircraft. A significant part of the aircraft’s weight consists of electrical conductors (power or signal). In 2015, a new concept of communication of avionics devices using wireless transmission (Wireless Avionics Intra Communication—*WAIC*) was introduced, from which a significant reduction in cabling weight is expected. However, this communication protocol operates in the same band as radio altimeters [6].

A more significant problem is the introduction of 5G communication technology (3.4–3.98 GHz), which, although it does not operate directly in the band reserved for ALT (4.2–4.4 GHz), causes interference based on various reports and observations. The 5G network is open for licensing from December 2020. This issue is currently the subject of research and more studies, and no specific conclusions have yet been drawn, only recommendations [6,7]. However, the results presented in the report [3] reveal a major risk that 5G telecommunications systems will cause interference to onboard radar altimeters on all types of civil aircraft (commercial airplanes, business jets, regional aircraft, and general aviation airplanes and both transport and general aviation helicopters. Further work, like [6], shows that there is no interference of 5G technology with radio altimeters, which can be confusing. The implementation of 5G services in individual countries, different industry standards or regional government regulations may change in the future. This can lead to the change of parameters in such a way that they can start to cause interference (even if previous operation was without problems) to radio altimeters or, the opposite, stop causing previously observed interference. This problem must be examined with state-of-the-art regulations. In addition, testing the adverse effects of 5G on ALT in real conditions is very demanding and even risky [8]. Although the method of measurement of ALT accuracy presented in this article is focused on evaluating the methodological error Δ*H*, it could be a suitable alternative for this area of research. The measurement chain can be supplemented with sources of 5G signals. The direct radio altitude reading, together with methodological error evaluation (with 5G signal sources turned on and off), can help to investigate if the radio altitude measurement is affected or not.

Usually, ALT is used for measurements of altitude up to 750 m above the terrain (the ground). For higher altitude and flight level measurements, barometric altimeters, operating on a different principle of operation, are used. Radio altimeters have many advantages, but they are associated with technical limitations related to the accuracy of altitude measurement. Current *ALT* types most often measure height with an accuracy of ±0.30 m to ±0.75 m. This value of measurement inaccuracy is related to the error of the height measurement method itself, which uses frequency modulation. This is the so-called methodological error of *ALT* measurement (Δ*H*) [9]. The motivation for creating the presented research was the fact that the theoretical values of the accuracy of the height of the measurement above the ground have been given as final values for the relevant type of *ALT*. Nevertheless, the errors resulting from the design and circuit solution of *ALT* must be added to the determined value of the methodological error of height measurement [9]. It is also necessary to consider the purpose of using the device—installation on a dynamically moving object. In such a case, these are mainly Doppler effect errors, the difference in the frequency of the difference signal, the fluctuation of the received signal, the flight dynamics of the aircraft, the delay of the *ALT* circuits and the parasitic amplitude modulation of the *HF* signal (Figure 1).

The effect of these errors can be seen in some flight modes or only over a specific type of land surface. In this case, the reported values of height measurement accuracy are numerically lower than their theoretical value (from ±0.30 m to ±0.75 m), and some long-term used *ALTs* show an unstable value of the methodological error. For this reason, the operation and stability of the function of the additional output signal processing circuits are difficult to evaluate. All this has a negative effect on determining the value of the methodological error and, consequently, on the height measurement accuracy. We know from practice that, at present, it is not possible to evaluate a methodological error in the standard conditions of the operator [10,11,12,13].

The *FMCW ALT* method error is related to the value of the used frequency lift Δ*f*. The range of the frequency lift (Δ*f*) is related to the carrier frequency value (*f*_0_). Based on the above conditions, it can be stated that the first types of *ALT* had a methodological error Δ*H* = 2.2 m at Δ*f =* 17.0 MHz; *f_0_ =* 444.0 MHz. Using newer technologies, the *ALT* method error was reduced to Δ*H* = 1.0 m at Δ*f =* 25.0 MHz; *f*_0_ = 2.0 GHz. Currently used *ALTs* have a methodological error Δ*H* = 0.75 m at Δ*f* = 50.0 MHz; *f*_0_ = 4.4 GH*z*. These values of methodological error correspond to the respective values of *ALT* height measurement accuracies in the range ±Δ*H* = ±2.2 m; ±1.5 m; ±0.75 m.

This article aims to present a new method of experimental measurement of *ALT* methodological error, which is feasible in any laboratory conditions and allows consideration of other influences on the overall accuracy of radio height measurement using *ALT*.

## 2. Materials and Methods

Clarifying the theory of methodological error and its evaluation is key for understanding the method of determining the accuracy of radio altimeter (*ALT*) height measurement presented in the article. The whole research, development of methodology, and practical implementation of a measurement method are based on the practical experience of the authors [9,10,11].

The measurement error of radar altimeter RV-5 is ±0.75 m. This inaccuracy is caused by different measurement errors with varying shares in this total value. The largest share (70%) is from methodological error. The second largest share is from frequency modulation parameter instability (20%). The third share is from aircraft dynamics error (6%). The last share is from the combination of parasitic modulation and the error of external conditions (4%). If the measurement error value of ±0.75 m is 100%, the individual values of sub-errors can be expressed in numerical values like in Table 1.

The main measurement errors of radio altimeters can be briefly explained as follows:

- Methodological error.
- Frequency modulation parameter instability error includes errors caused by frequency stroke instability, modulation frequency instability and frequency modulation nonlinearity. This error is suppressed by the altimeter radio automatic tuning system.
- Radio altimeter dynamic error—error caused by radio altimeter inertia (t ≈ 0.5 s); error due to aircraft flight dynamics (perpendicularity of the antenna radiation axis to the reflecting surface/ground/depolarization panel).
- Remaining measurement errors can be explained as follows:
- Radio altimeter instrument error—it is possible to include here the error of evaluation of the frequency difference and the error of the altitude indication (this error is unique for each device).
- Doppler effect error—generated with each dynamic altitude change (caused by increasing or decreasing flight altitude).
- Fluctuation error—is caused by the random nature of the reflected signal and the effect of the internal noise of the receiver.
- Shift error—the error is caused by the shift of the energy spectrum of the difference signal to the right along the frequency axis, towards higher frequency frequencies.
- Error due to external conditions—this is an error caused by a change in the characteristics of the environment in which the radio altimeter operates.
- The parasitic modulation error—is an error caused by a parasitic change in the amplitude of the received radio altimeter signal due to parasitic amplitude modulation or a parasitic signal.

As the methodological error will most affect the accuracy of measurement, we will focus on it in this article and explain this error in the following chapter.

### 2.1. The Principle of Operation of ALT and Its Methodological Error of Measurement

As mentioned in the introduction, the most used aviation radio altimeters are radar devices operating either in pulse mode or with frequency modulated continuous wave (*FMCW*) [1]. The *ALT* transmitting antenna emits a continuous sawtooth waveform modulated high-frequency carrier signal towards the ground during its operation. After the radiation and the subsequent reflection of the carrier signal from the ground, this signal is received by the receiving antenna. From there, it is processed to a balanced mixer, which is at the receiver’s input [9,10,11,12,13].

After travelling the path of the *aircraft*—*ground*—*aircraft*, the received signal is delayed by time delay *τ* (Equation (1)). This delay time of the received signal is proportional to the flight altitude of the aircraft $H_{r}$.

So:

(1) $$\tau =\frac{2H_{r}}{c}$$

where: *τ*—is the time delay; $H_{r}$—is the real measured radio altitude of aircraft over the ground; *c*—is the constant of the speed of light in a vacuum.

**Figure 2:**
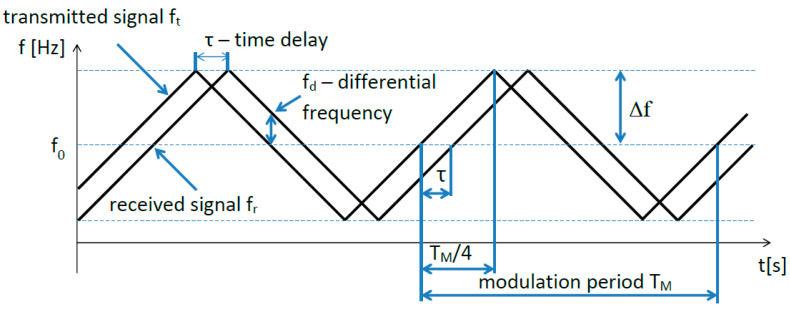
Graphic representation of dependence between transmitted and reflected signal.

In the equation, the height *H_r_* is doubled because the signal travels this distance twice—the signal passes from the aircraft to the ground (downwards) and from the ground to the plane (backwards). The radiated, reflected, and received signal still has the same frequency even after a time delay *τ*. The received signal meets the currently transmitted signal at the receiver’s input (in the mixer stage) [14]. However, the transmitted signal has shifted from the original frequency of the received signal during the time *τ* because of the frequency modulation (Figure 2). The instantaneous frequency difference $f_{d}$ between the transmitted signal $f_{t}$ and the received signal $f_{r}$ is proportional to the measured height $H_{m}$ (Equation (2)).

(2) $$H_{m}\;\approx \;f_{d}=\left|f_{r}-f_{t}\right|$$

where: $H_{m}$—is the measured height of aircraft over the ground; $f_{d}$—is the instant frequency difference between transmitted and received signal; $f_{t}$—is the frequency of the transmitted signal; $f_{r}$—is the frequency of the received signal.

There is a difference between the actual radio flight altitude $H_{r}$ and the measured altitude $H_{m}$ caused by the method of indirect altitude measurement by the time delay of the signal *τ*. Considering this measurement method, the evaluation procedure is as follows. The actual measured height $H_{r}$ is proportional to the time delay *τ*. The time delay itself is measured indirectly by means of a difference frequency $f_{d}$ which is proportional to the difference between the high-frequency carrier frequency of the transmitted signal $f_{t}$ and the received signal $f_{r}$. The carrier frequencies of both the transmit and receive signals are in the GHz range. The frequency difference is then stepped-down and transformed from the GHz range to the kHz range. Because of the frequency modulation of the carrier signal, the frequency spectrum of the difference signal, which carries the information about the measured height, is complex—it is therefore not possible to directly evaluate the difference frequency. The evaluation takes place by adding the number of impulses *N* per unit of time. This number of impulses carries information about the measured height $H_{m}$. A simplified mathematical equation (Equation (3)) can express this indirect way of measuring height.

(3) $$H_{m}\approx N\approx f_{d}=\tau =H_{r}$$

where: *N*—is the number of impulses per unit of time.

It seems that there is a linear relationship between the actual radio altitude and the measured altitude of the aircraft. This can be expressed by the constant *K*. In this case, it would be possible to write:

(4) $$H_{m}=KH_{r}$$

where: *K*—is the function of transformation changes of signals.

Where the value of *K* would correspond to the function of transformation changes of signals *K = f* (*τ*, *f_d_*, *N*). However, a linear relationship exists only between the time delay of the received signal and the actual altitude. This fact can be expressed by an appropriate adjustment of the mathematical equation (Equation (1)).

(5) $$\tau =\left(\frac{2}{c}\right)H_{r}$$

In this case, the value of the constant *(2*/*c)* is so small that the measurement of the time delay was not feasible for many years when measuring the height. This problem was solved by the American inventor *E.H. Armstrong*, who used frequency modulation of the transmitted signal to measure altitude. His patent transformed the problem of measuring a very short delay time *τ* when measuring small heights into evaluating low values of the difference frequency $f_{d}$.

This transformation can be written mathematically using the so-called radio altimeter constant—$K_{alt}$. This mathematical relation represents the basic equation of the radio altimeter (Equation (6)) [15].

(6) $$f_{d}=K_{alt}\;H_{r}=\frac{8\;\Delta f\;f_{m}}{c}\;H_{r}$$

where: Δf—is the frequency lift; $f_{m}$—is the modulation frequency.

**Figure 3:**
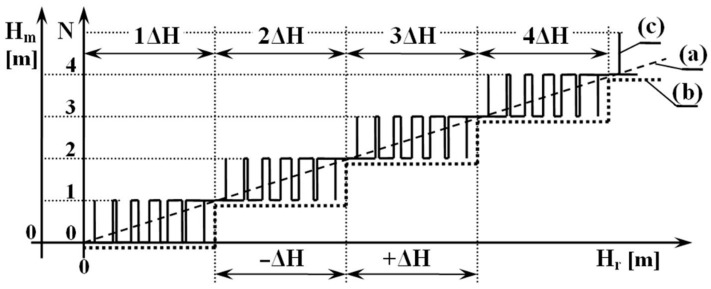
The structure of creation of methodological error of radio altimeter.

For a better idea, current *FMCW* radio altimeters use the following basic modulation parameters: Δ*f* = 50.106 Hz; $f_{m}\;$= 150 Hz; *c* = 3.108 ${\mathrm{ms}}^{-1}$. In this case, the numerical value of the radio altimeter constant is $K_{alt}\;$*=* 200. This dependence can be expressed graphically by a line, while the value of $K_{alt}$ expresses the angle of inclination of the line. The mentioned linear course can be seen in Figure 3, the dashed line marked by the letter (a).

According to the stated relationship and at the stated modulation parameters, it is possible to measure the height in steps of 0.5 cm. When changing the altitude with this step, the difference frequency changes by 1 Hz. For minor altitude changes, the mathematical result is not a solid numerical value of the frequency—for this reason, it is not possible to measure a minor altitude change. From the point of view of practical measurement of the aircraft’s height above the ground (altitude), we could consider such a measurement process (with a discretion step of 0.5 cm) to be ideal.

However, the dependence of height measurement is not linear. The problem is related to the principle of frequency modulation. The smallest change in the height of 0.5 cm means an increase in the frequency of 1 Hz, but only within one modulation period $T_{m}\;$(Figure 2). Since the modulation period is repeated *x* times in the rhythm of the modulation frequency $f_{m}$, this means that the number of impulses per unit time *N* is $f_{m}\;$ times larger [15].

For example, if the current *FMCW ALT* uses the modulation frequency of $f_{m}$ = 150 Hz, then the discrete (step) increase in the difference frequency ∆f will also be 150 Hz. In terms of the principle of the frequency modulation function, this means that if the height range is 0.5 cm per one modulation period, then the smallest change in height at 150 modulation periods will be 0.5 cm × 150 = 75 cm = 0.75 m.

The dependence of the value of the difference frequency on the real height will have a stepped course—as can be seen in Figure 3, a dotted graphic course (b). This minimum altitude range that a radio altimeter can evaluate by measuring is called the critical altitude. This critical altitude, which affects the accuracy of *FMCW ALT* measurements, is based on the altitude measurement method itself—technically called the methodological error of the radio altimeter.

The methodological error is marked ∆*H* and is inversely proportional to the value of the frequency lift ∆*f* and is defined by the equation:

(7) $$\Delta H=\frac{c}{8\;\Delta f}$$

where: ΔH—is the methodological error of height measurement.

The measurement error can be most easily explained by the stepped relationship between the actual and the measured height. As mentioned, current radio altimeters have a method error value of ∆*H* = 0.75 m. This value is defined theoretically, as it is not possible to measure this course in the form in which it is described. This is due to the fact that two types of height impulses are involved in generating the difference frequency $f_{d}$.

The first type is the so-called constant impulses, the number of which corresponds to the measured height. The second type of impulses are the so-called transient impulses, which create a methodological error of the radio altimeter.

The number of constant impulses increases in steps as the flight altitude increases—at 150 Hz, with an altitude step (critical altitude) of 0.75 m. As the flight altitude decreases, the number of constant impulses decreases in steps—after 150 Hz with a height step of 0.75 m.

The number of unstable impulses increases and decreases alternately as the flight altitude (height) increases, after 150 Hz over the entire critical altitude range. As the flight altitude decreases, the number of transient impulses also increases alternately and decreases by 150 Hz over the entire critical altitude range. This ambiguity in the number of unstable impulses creates the mentioned methodological error of height measurement. The dependence of the value of the difference frequency *∆f* on the real height, as the sum of constant and unstable impulses, will have a variable step course. This is shown in Figure 3 by course (c).

Figure 3 graphically expresses the altitude (height) change after only one impulse within one modulation period. At a modulation frequency of 150 Hz, 150 impulses are generated and extinguished on the *FMCW ALT* per unit time.

As already mentioned, and also graphically represented (Figure 3, graphical course (c)), the radio altimeter evaluates the obtained data on the changing altitude over the entire range of the measured altitude (height) constantly, through a changing number of impulses of frequency difference, in the range of ±150 Hz. The mentioned change of frequency is practically realized during (change) of height measurements, but the exact values of ±150 Hz are only at the theoretical level. The reason is the fact that one exact discrete value is not created even when measuring a stable height value—even when the *ALT* high-frequency path is closed with a coaxial cable. Several factors cause this phenomenon:

- the number of impulses changes as soon as the height changes by 0.5 cm;
- for the frequency 4.4 GHz, the wavelength is λ = 68 cm—in this case, the value 0.5 cm represents only 2.65°;
- when evaluating $f_{d}$ at the level of *HF* signals $\left|f_{r}-f_{t}\right|\;$ the number of impulses changes already at the level of a small phase change;
- the phase change is caused by the frequency modulation process itself;
- a small phase change is also caused by a small instability of the repetitive sawtooth waveform of the *HF* signal;
- in real altitude measurement, a change in the terrain quality under a flying aircraft causes a change in the load for the *RF* transmitting circuits—this changes the course of the modulated *RF* signal.

When we add to these negative factors the fact that the number of impulses can change from one modulation period to another during the height measurement, the number of impulses can also change with values lower than 150 Hz. This affects the height measurement to such an extent that it is very difficult (even impossible) to record the experimental measurement results as described and as shown in Figure 3, graph (c). For this reason, it is difficult, if not impossible, to determine the exact value of the *FWCW ALT* methodological error by such experimental measurements [16].

### 2.2. The Methods for Evaluating the Methodological Error of the Radio Altimeter

There are several ways in which it is possible to evaluate the methodological error of a radio altimeter which take into account the existence of stable and unstable impulses in the frequency difference. They are most often based on known technical parameters of *ALT*. They are:

- method of direct calculation of the methodological error;
- method of defining of the height impulses;
- method of using of the simulation program;
- method of classical experimental measurement;
- method of the laboratory experimental measurement [16].

#### 2.2.1. The Method of Direct Calculation of the Methodological Error

This is the best known and most straightforward way to determine a methodological error using a mathematical equation (Equation (7)). This relationship directly defines the value of the methodological error according to stable height impulses. However, it does not take into account the involvement of unstable impulses in the generation of the frequency difference [16,17].

#### 2.2.2. The Method for Defining of Height Impulses

This is a convincing but lengthy method due to the need to manually create a graph of height dependence. Mathematical relations are used to determine the height values for the creation, extinction, and mutual overlap of the height impulses. This method is based on the mathematical analysis of the *ALT* theory [16], on the basis of which mathematical relations are defined for determining the height of each height impulse $H_{cre}$ (Equation (8)) and subsequently also the extinction height of the given height impulse $H_{ces}$ (Equation (9)).

(8) $$H_{cre}=\frac{\lambda_{0}}{8}\;\frac{2k-1}{1+\xi }$$

(9) $$H_{ces}=\frac{\lambda_{0}}{8}\;\frac{2k-1}{1-\xi }$$

where: $H_{cre}$—is the creation of height impulse; $H_{ces}$—is the extinction of height impulse; k—is the number of the impulse; $\lambda_{0}$—is the wavelength of the carrier wave; ξ—is the relative value of frequency lift $\xi =\frac{\Delta f}{f_{0}}$.

The height range of the duration of each height impulse $H_{dur}$ is determined as the difference between the height of the extinction $H_{ces}$ and the height of the origin $H_{cre}$ of the respective impulse:

(10) $$H_{dur}=H_{ces}-H_{cre}=\frac{\lambda_{0}}{4}\left(2k-1\right)\frac{\xi }{1+\xi^{2}}$$

where:$\;H_{dur}$—is the height range of duration of each height impulse; $f_{0}$—is the carrier frequency.

**Figure 4:**
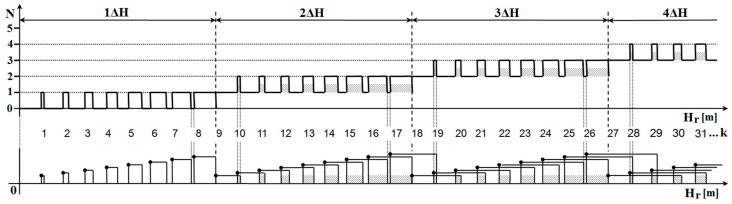
Mathematical analysis of methodological error of radio altimeter [16].

It is clear from equation (Equation (10)) that the height range of the individual impulses increases with increasing sequence impulse number *k*. It is clear from Figure 4 that due to the increase in the height range, the individual height impulses overlap each other [15,16,17].

The number of overlapping height impulses determines the number of constant impulses. However, with increasing altitude, new height impulses are constantly generated, and consequently, the “old” height impulses, which were created at lower altitudes, constantly disappear. This constant process of creation and extinction of one height impulse takes place in the entire range of the measured height. In a certain altitude range, the mutual overlap of unstable altitude impulses increases so that one (another) stable altitude impulse is generated in the range ΔH. This height section represents the *ALT* methodological error.

#### 2.2.3. Method of Using of the Simulation Program

Based on the knowledge of the *FMCW ALT* theory, it is possible to create a simulation program for the creation of altitude impulses. After creating a simulation program, this is the simplest and fastest way to visualize the height dependence of the methodological error. This includes the generation of constant and unstable height impulses (*UAP*) involved in the generation of the difference frequency [16].

**Figure 5:**
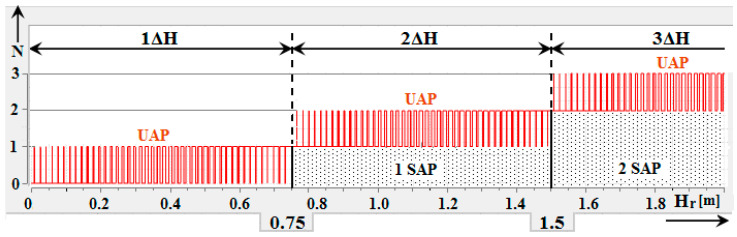
Simulation of methodological error of radio altimeter [16].

Figure 5 shows the simulation results for which input parameters were defined corresponding to the operating parameters of current radio altimeters. A problem related to a methodological error is highlighted in this simulation output. Only 44 unstable altitude impulses (*UAP*) exist in the range of 1ΔH measured height (0.0–0.75 m). In the range of 2ΔH measured height (0.75–1.5 m), there are another 44 unstable altitude impulses (*UAP*) but also one stable altitude impulse (*SAP*). In the range of 3Δ*H* measured height (1.5–2.25 m), there are another 44 unstable altitude impulses (*UAP*) but also two stable altitude impulses (*SAP*). The number of unstable height impulses $N_{u}$ in the range ΔH can be determined by (Equation (11)) [15]:

(11) $$N_{u}=\frac{f_{0}^{2}-\Delta f^{2}}{2\;f_{0}\;\Delta f}$$

where: $N_{u}$—is the number of unstable height impulses.

With increasing altitude, the situation is repeated so that every 0.75 m 44 unstable altitude impulses (*UAP*) are generated, and one stable altitude impulse (*SAP*) is always added.

#### 2.2.4. The Method of Classical Experimental Measurement

From a technical point of view, this is the most demanding method, but it is possible to achieve the most reliable result in determining the value of the methodological error. This method represents an experimental measurement of methodological error in laboratory conditions. The advantage of this method is that the experimental results include the error of the measurement method and the error introduced by the technical and circuit solution of *ALT* in the evaluation of height, which can be unique for each device [18,19].

The experimental measurement itself cannot be performed in a routine laboratory or other non-adapted enclosed space [20]. The reason is the sensitivity of *ALT* to parasitic reflections of the high-frequency signal. As a result, it is not possible to measure the distance (height) from the prepared reflecting surface in enclosed spaces with a radio altimeter, nor to evaluate its methodological error. The given method of classical experimental measurement can be realized only in a specially adapted attenuation chamber, using antennas in the high-frequency path, or in an ordinary laboratory, using different lengths of coaxial cables, which serve as a substitute for changing the distance of the high-frequency route.

Despite all efforts, this method seems relatively inaccurate. In addition, it is not possible to use this method on new types of *ALT* operating at 4.4 GHz. This is due to the threat of damage to the *RF* circuits due to frequent connections and disconnections of the *RF* route [21].

**Figure 6:**
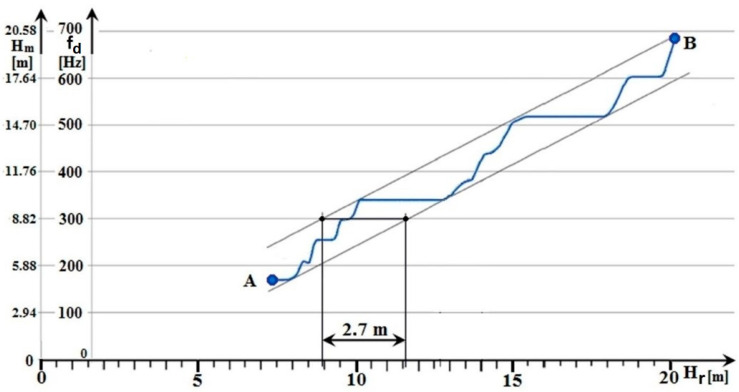
The results of classical measurement of methodological error of radio altimeter.

The result of the *ALT* height measurement, using a regular discrete change in coaxial cable length, is shown in Figure 6.

The methodological error measurement, the graphical result of which is shown in Figure 6, was performed on an older type of *ALT*. This radio altimeter operates with a carrier frequency of 444 MHz and a frequency lift of 17 MHz. At this frequency lift value, the methodological error is 2.2 m, and the measurement error is ±2.2 m. The range of the measured height of 12.6 m was realized by changing the length of the coaxial cable with a step of 0.2 m. This represented *63* measurement steps.

The measured (tested) older type of *ALT* was not actively used on the aircraft for a long time. The maintenance with compliance of the manufacturer was performed on it before the measurement. Nevertheless, the *ALT* measurement showed an excessive error rate. Specifically, the measurement error value was 0.5 m higher, i.e., ±2.7 m, compared to the measurement error specified by the manufacturer (±2.2 m). The disadvantage of this measurement method is that it is not possible to evaluate the classical methodological error (as was presented in Section 2.2.3).

In view of the larger measurement error of the radio altimeter (±2.7 m instead of the theoretically assumed measurement error *ALT* ± 2.2 m), it can be stated that the measured (tested) *ALT* did not meet the requirements for integration into active usage on aircraft. This is despite the fact that the results of the checked parameters according to the maintenance did not indicate this. It follows from the above that, in addition to checking the parameters themselves, it is appropriate to check the value of the methodological error at certain time intervals. However, the mentioned measurement is not performed in the technical operation as standard—due to the complexity of the technical provision of the measurement.

#### 2.2.5. The New Method for Experimental Laboratory Measurement

A control measuring apparatus is used during the implementation of prescribed work (maintenance) of aircraft equipment, such as a radio altimeter, which takes place in laboratory conditions. This apparatus allows you to check and evaluate important *ALT* parameters. It also allows you to imitate the height—but only on selected discrete values, without the use of antennas. It is impossible under standard laboratory conditions to test the operation of a radio altimeter using antennas and a reflecting surface located at a certain distance from the antennas to imitate the ground surface under a flying aircraft. The reason is undesirable parasitic reflections of the *HF* signal from various metallic as well as non-metallic objects and surfaces in the laboratory—these cannot be suppressed in the usual way, and *ALT* cannot eliminate them. Measurement of *ALT* in laboratory conditions with the inclusion of a high-frequency path (with antennas) is only possible in an anechoic chamber. However, this chamber has limiting dimensions for the object’s size to be tested/measured. Due to its high purchase price and overall financial demands, it is mainly part of the top workplaces/laboratories.

The article primarily eliminates the mentioned shortcomings by a new *ALT* measurement system, which can eliminate its parasitic reflections. When measuring the distance (altitude of the aircraft), a reflector panel with a change in the polarization of the radio waves is used. This panel is placed into the *HF* path between the transmitting and receiving antenna *ALT* [22,23,24].

**Figure 7:**
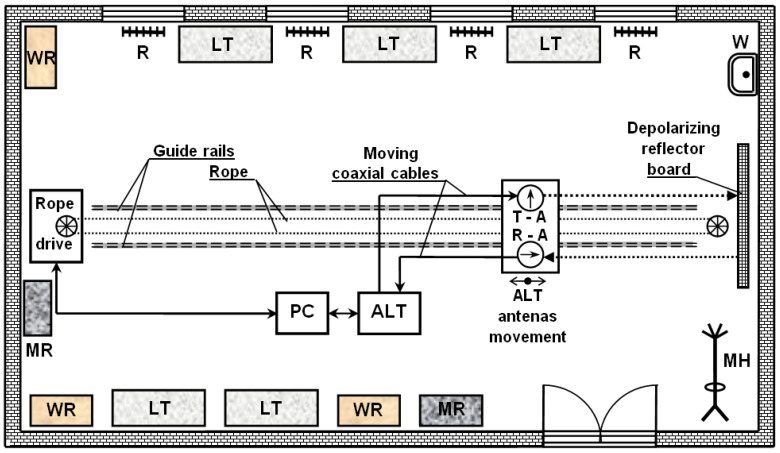
Illustration figure of the layout of a measuring workplace in a laboratory, where: **LT** is a metal laboratory bench; **MH** is a metal hanger; **MR** is a metal case; **R** is a radiator; **W** is a sink; **WR** is a wooden cabinet; **T**-**A** is a transmitting antenna; **R**-**A** is the receiving antenna.

The principle of distance measurement using a radio altimeter in laboratory conditions is as follows. For example, if a vertically polarized wave is used in the transmission, then all parasitic reflections from surrounding objects and laboratory surfaces also have vertical polarization. The essence of the elimination of parasitic reflections lies in the rotation of the polarization of the initially transmitted signal on the patented reflection depolarisation panel by 90° [22,23,24]. The reflected signal used to measure the distance then has horizontal polarization. In this way, the useful distance measurement signal is significantly differentiated from the useless parasitic reflections in the laboratory. Furthermore, the receiving antenna picks up only the reflected signal from the reflective depolarisation panel in this distance measurement system. This changes the vertical polarization of the transmitted signal to horizontal polarization. In this way, the reception of parasitic reflections of the transmitted *ALT* signal, which is fully functional in these conditions, is significantly reduced (Figure 7).

**Figure 8:**
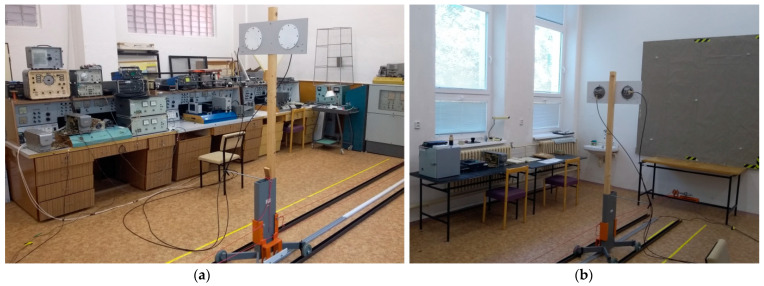
Different views of the laboratory and the measuring workplace of the radio altimeters, where figure (**a**) illustrates the movable antenna system connected to the examined ALT and figure (**b**) represents the antenna system using the depolarisation reflection panel.

The structural arrangement of the measuring workplace (the laboratory) is adapted to the mentioned method of measuring of methodical error of the *ALT* (type of *FMCW*). The development of the presented laboratory (Figure 8) was initially being carried out for pedagogical support of the education of students in the field of radio altimeters at the Faculty of Aeronautics of the Technical University of Košice.

Subsequently, this laboratory became a research institute for researchers in selected topics of avionics systems.

In Figure 8a it is possible to see the measuring station of the *ALT* system together with the surrounding several metal objects. Figure 8b shows a patented depolarisation panel [22,23,24] with dimensions of 2.2 m × 2.2 m, which changes the polarisation and also serves as a reflecting surface (simulation of the terrain under a flying aircraft). A black U-shaped iron profile can be seen on the floor of the room for the directional guidance of the *ALT* antenna stand—it ensures the stability of the direction of the antennas in the horizontal plane during their movement. The white-grey E-shaped plastic profile was used to stabilise the direction of the antennas in the vertical plane on the depolarisation panel as they moved during the measurement. The yellow I-shaped plastic measuring band served for a quick visual orientation of the antennas’ continuous position and for determining the start and endpoint of the measurement.

This figure (Figure 8a,b) demonstrates a significant fact—quality and accurate height measurement can be realized using our method in a standard laboratory. In the illustrated case, the measurement was performed at a frequency of 4.4 GHz in a laboratory equipped with various metal objects. Without the use of a patented depolarisation panel, it would not be possible to perform such a measurement, even in an empty laboratory [22,23,24].

The radio altimeter type *RV*-*5* and later *ALT 55* were chosen for experimental measurements. This radio altimeter (*RV*-*5*) was excluded from active air traffic, although it was functional—this met the experiment’s needs, as it was possible to implement the necessary intervention.

The chosen *RV*-*5* radio altimeter operates in *FMCW* mode with the following parameters. The carrier frequency is *f*_0_ = 4.4 GHz; frequency modulation *f_m_* = 150 Hz has a sawtooth waveform; frequency lift is *±*Δ*f =* 25 MHz; critical height corresponds to Δ*H* = 0.75 m, and measurement accuracy is up to ±0.75 m. For the needs of experimental measurements in the laboratory, a suitable pad was made for its fastening, connection, and control. Subsequently, the necessary direct current sources (28 V DC) and alternating voltage (115 V/400 Hz AC) were connected. Before use, the functional operation of the radio altimeter was checked by a specified control and measuring apparatus (*KPRV5*). The original antennas of the radio altimeter were used—their location was on a unique mobile stand, and they were mounted on a metal imitation of a small fuselage area. The power supply of the antennae was achieved by means of flexible coaxial cables, which moved freely on the ground during manipulation (movement of antennas during measurements). The length of coaxial cables was the same as it is in real aircraft/helicopters to achieve almost the same conditions as in actual operation. However, this length of coaxial cables will result in so-called residual height, which must be compensated by the radio altimeter. i.e., if we add 10 m of coaxial cables to the transmitting or receiving line, it will add 5 m of radio altitude to the measurement. To keep the cables’ position (near the antennas), a simple holder was designed and constructed, ensuring a stable place for them during the measurement. The drive of the mobile stand on metal rails was realised using a nylon rope, which was driven by a stepper motor with a slow transmission to a larger drive wheel. The rope was tensioned by a driven wheel, which was located on the other side of the laboratory.

**Figure 9:**
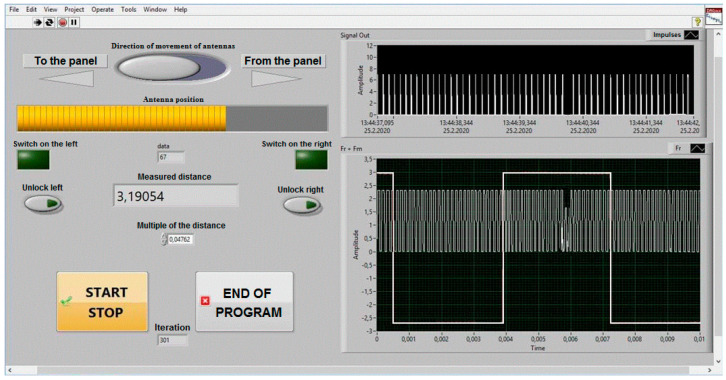
The virtual control panel of the measurement system.

Sensing the current position of the antennas, their speed of movement and the range of measured height was controlled by a computer and using appropriate auxiliary circuits. The guide mechanism stabilised the movement of the mobile stand in the horizontal and vertical planes. Control of height (distance) measurements in the laboratory, control of antenna movement and display of their position, including recording of measured data, was performed with a computer using a *DAQ* control device from National Instruments, controlled by a program in the *LabVIEW* environment. A virtual control panel (Figure 9) was implemented on this platform, which controlled and sensed the position of the *ALT* antennas. The virtual control panel allows you to control the speed and direction of movement of the mobile antenna stand and records the dynamic change in height measured by a radio altimeter. The output data of the measured height from the *ALT* was connected to the computer using the *DAQ* converter *NI USB DAQ 6216*. In this way, it was possible to start and stop the recording of the measured data of the modulation frequency *f_m_* and the difference frequency *f_d_*. This includes added information about the distance of the *ALT* antennas from the reference reflecting surface.

The right side of the control panel consists of displays—oscilloscopes, which show the measured signals of the radio altimeter. They are needed for the graphical display of its methodological error. To graphically display the critical height Δ*H*, from which is determined the methodological error *ALT* (*±*Δ*H*), it is necessary to know the number of impulses N of the frequency difference *f_d_*, within one modulation period *T_M_*, and the modulation frequency *f_m_*, where $T_{M}=\frac{1}{f_{m}}$. For this reason, at the right bottom (Figure 9), there is a recording of a low-frequency rectangular signal with a modulation frequency of 150 Hz. The result of *ALT* activity is shaping the difference frequency with the value of units of kHz to tens of kHz, depending on the measured high. The rising edges of this rectangular impulse of the modulation period determine the point for starting the counting of impulses of the frequency difference. It can be seen from this graphic waveform (Figure 9) that the modulation signal overlaps a difference signal of approximately 3 kHz. Impulses are formed from the difference frequency, the number of which (per modulation period) determines the measured height value. The impulses shaped in this way are shown at the top of the record (Figure 9).

## 3. Analysis of Results of Measurement of Methodological Error

**Figure 10:**
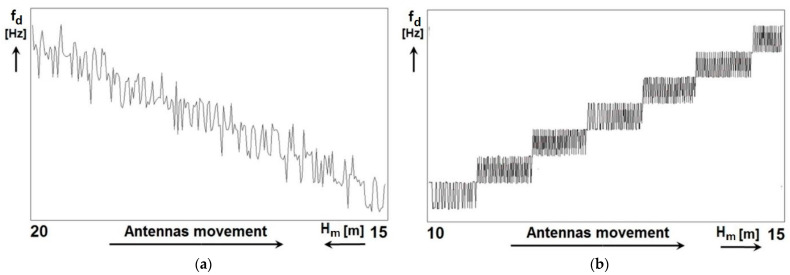
ALT methodological error measurement results. (**a**) is height reduction measurement at a rate of 0.56 ${\mathrm{ms}}^{-1}$; and (**b**) is height increase measurement at a rate of 0.28 ${\mathrm{ms}}^{-1}$.

The results of measuring the method error of the radio altimeter by the proposed new method can be seen in Figure 10. The article presents three types of measurements that differ in the speed and direction of antenna movement.

In terms of speed, the highest speed was used in the first measurement (0.56 ${\mathrm{ms}}^{-1}$). In the second measurement, the speed was medium, i.e., 0.28 ${\mathrm{ms}}^{-1}$, and in the third measurement, the speed was the lowest, 0.14 ${\mathrm{ms}}^{-1}$. The definition of the speed of movement of the *ALT* antennas, namely *high*, *medium* and *low* speed, is chosen to take into account the dimensions and technical capabilities of the laboratory. In terms of the direction of movement of the antennas, this was realized by reducing the height (distance) by bringing the antennas closer to the polarizing panel [22,23,24]. Or, conversely, by increasing the height (distance) by moving the antennas away from the polarizing panel. Considering the laboratory’s length (7 m) and the necessary technical equipment, it was possible to measure the height (distance) in the range of 5 m.

Figure 10a shows the measurement result when the distance (height) was reduced (simulation of descent) in the range from 20 m to 15 m. In Figure 10b the result of the measurement when the distance (height) was increasing (simulation of the climb) in the range from 10 m to 15 m. In the performed measurements, the minimum height is 15 m in Figure 10a and 10 m in Figure 10b, which is the so-called residual height.

The residual height is formed by the length of the coaxial cables of the antennas and the minimum distance of the antennas from the polarizing panel, at which the measurement always stopped. The reduction of the value of the residual height from 15 m to 10 m was realized by changing the length of the coaxial cables of the antennas.

The representation of both records in Figure 10 represents the actual measurement results, and they are for illustrative purposes. They show the difference in measurement at two different speeds and at two different directions of antenna movement. All three presented measurements were performed on one and the same *FMCW* radio altimeter type *RV*-*5*.

When processing the differential signal of the radio altimeter as information about the measured altitude, the following steps are performed during processing:

- harmonic difference signal amplification and frequency filtering;
- amplitude trimming and shaping rectangular impulses from the difference signal;
- deriving edges and generating impulses from the rectangular shape of the difference signal;
- detecting and removing single polarity impulses;
- impulse integration (voltage shaping), which is proportional to the measured height.

For graphical recording and visualization of the method error of the radio altimeter by measurement, it was necessary to use a detected pulse signal of one polarity, the number of impulses of which corresponds to the difference frequency. For its optimal display, it was necessary to evaluate the number of impulses (generated as described above) during one modulation period. In the presented new method, the average value of the number of impulses in one period was evaluated from the measurement of the number of impulses in ten periods. For this reason, each measurement output lasted 10 modulation periods—as measuring the number of impulses in only one period did not work. This fact affected the quality of the methodological error display depending on the rate of dynamic height change. This phenomenon can be compared between Figure 10a,b.

**Figure 11:**
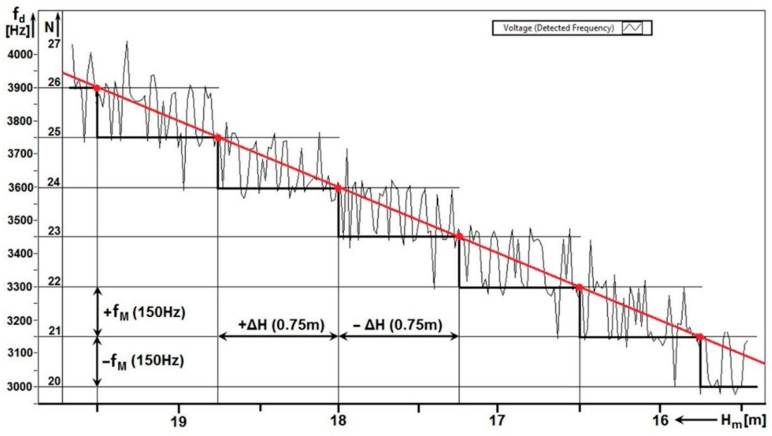
Analysis of methodological error of radio altimeter ALT measured at speed of 0.56 ${\mathrm{ms}}^{-1}$.

Figure 11 shows one of the results of measuring the methodological error at a higher rate (speed), i.e., 0.56 ${\mathrm{ms}}^{-1}$. It is evaluated as the average of the value of 10 $T_{m}$.

In each period, the number of impulses is represented by an integer. However, when measuring the number of impulses as an average value from ten periods, the result may not be an integer. The value of the integer always changes between two heights ΔH. In Figure 11, the transitions are represented by red dots. When recording the number of impulses *N*, their numerical value is proportional to the measured height *H*. In the height range, towards the higher height H+ΔH, the number of impulses every 9.4 mm changes in the range (N; N+1; N; N+1;…). In total, the number *N* can be changed up to eighty times in the ΔH range. In the height range towards the lower height H−ΔH, the number of impulses varies in the range *(*N; N−1; N; N−1;…). These changes in the value of the measured height N±1 in the range H± ΔH represent a methodological error of *ALT*. Therefore, the height measurement is not continuous but discrete, in the range of ±ΔH.

When measuring altitude, the radio altimeter evaluates the total number of impulses as a difference frequency $f_{r}\;$ proportional to the measured altitude. After integration, the difference frequency is transformed into a voltage $U_{H}\;$ proportional to the height. This proportional voltage is fed to the altitude indicator, which serves as primary information for the pilot of the aircraft or helicopter. And this form of *DC* voltage is intended for other systems (autopilot, anti-collision system, etc.). Since the number of impulses N is evaluated within the measurement of the methodological error, it is possible to combine this data with the difference frequency $f_{d}\;$ and the measured height $H_{m}$ by means of a simple mathematical transformation.

For each radio altimeter, it is possible, based on the parameter—the total frequency lift Δf, to calculate its methodological error ΔH according to the mathematical relation (7). In the case of the measured radio altimeter, this is the mentioned value of 0.75 m.

When each measured value of the number of impulses *N* per modulation period $T_{m}$ is multiplied by the modulation frequency $f_{m}$, we get the value of the difference frequency $f_{d}$.

(12) $$f_{d}=Nf_{m}$$

For example, at N=22 and $f_{m}=150\;\mathrm{Hz}$ is the $f_{d}=3300\;\mathrm{Hz}$. In this way, it is possible to determine the scale of the vertical—frequency axis of the graph. In general, the basic equation of radio altimeters is defined for *ALT*, which defines the linear relationship between the measured height $H_{m}$ and the difference frequency $f_{d}$ by means of the proportionality constant K.

(13) $$f_{d}=\left(\frac{8\;\Delta f\;f_{m}}{c}\right)H=KH_{m}$$

For the ALT on which the measurement was performed, the proportionality constant has the value of K=200. Using the proportionality constant, it is possible to determine the measured height from the difference frequency. For example, for $f_{d}=3300\;\mathrm{Hz}$, the is equal to $H=\frac{f_{r}}{200}=\frac{3300}{200}=16.5\;m$. In this way, it is possible to determine the horizontal scale, i.e., the height axis of the graph.

The quality and accuracy of recording the methodological error of the radio altimeter ΔH depend, among other things, on the height sensing speed, i.e., on the speed of movement of the antennas during the measurement.

At a high measurement speed of $0.56{\;\mathrm{ms}}^{-1}$, the ALT antennas pass the critical height ΔH=750 mm in 1.33 s. In the time range of 1.334 s the 200 modulation periods will pass. When determining the average value of the number of impulses N in each of the ten modulation periods, a small number (approximately 20) of measurements will be recorded in the altitude range ΔH at the time 1.334 s. An indistinct graphical representation of the methodological error due to the small number of recorded measurement results can be seen in Figure 10a and Figure 11.

**Figure 12:**
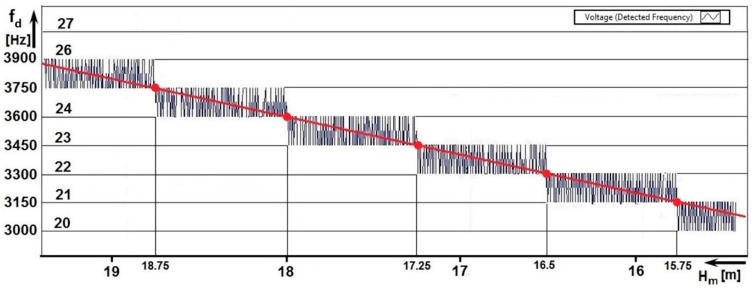
ALT method error analysis measured at speed of $0.14{\;\mathrm{ms}}^{-1}.$

At lower speeds, the number of measurement records is larger, and the graphical representation of the methodological error is much better. At a mean speed of $0.28{\;\mathrm{ms}}^{-1}$, the antennas pass the critical altitude ΔH in 2.667 s, which represents $400\;T_{M}$. In this way, a larger number of measurements will be recorded in the ΔH range—approximately 40 measurements (Figure 10b). At a low speed of $0.14{\;\mathrm{ms}}^{-1}$, the antennas pass the critical height ΔH in 5.334 s, which is $800\;T_{M}$. In this way, a large number of measurements will be recorded in the ΔH range—of approximately 80 (Figure 12).

From the graphical waveforms, it is also possible to evaluate the *linearity* of the height dependence measurement by simply translating the stepped line with a straight line. The *linearity* of the altitude measurement process is an important indicator of the *accuracy* of the altitude measurement (for example in a scenario where the aircraft is low above the runway at the take-off stage or the final approach). The second evaluated parameter is the *value of the critical height* Δ*H* when measuring the same radio altimeter for determining the basic parameter—methodological error.

By evaluating these two parameters of the radio altimeter (*linearity of the course* and the value of the *methodological error)*, the measurement accuracy, technical condition, and quality of the radio altimeter can be evaluated. Both parameters can be concentrated in one graph (Figure 11).

With the help of graphical evaluation, it is possible to register any inaccuracy in the setting of its parameters or imperfections in the operation of any circuit. If the radio altimeter shows even a slight discrepancy with the required parameters, its course of the increase of the difference frequency will not be linear, and in terms of methodological error, it would not be symmetric. In this sophisticated but simple way, it is possible to compare qualitative indicators not only of one type of radio altimeter but also different types of radio altimeters with each other.

All control measurements were performed on the same type of radio altimeter RV-5 but on four different units. The radio altimeters were controlled following manufacturer technical notes and with original control equipment KPRV5. After the checkup, we performed a total of 5 control measurements for each unit by this new method for evaluation of methodological error (total of 20 measurements). The results showed a difference of methodological error of 1.5% between the four units (Table 2).

**Figure 13:**
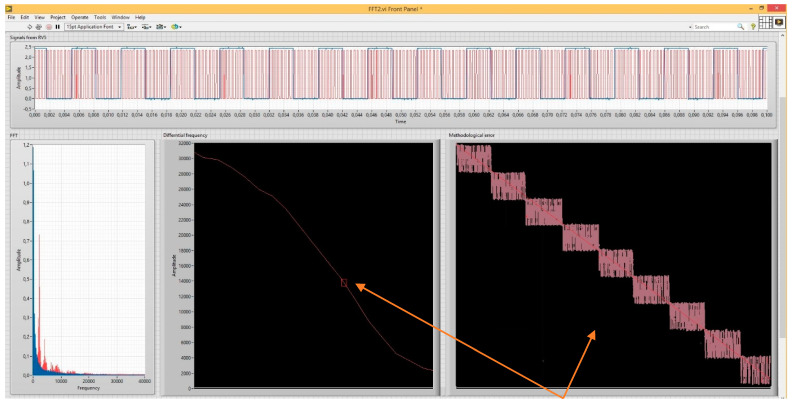
Measurements of methodological error in outside environment. The arrow points on examined 5 m radio altitude part from 250 m measurement range.

The main objective for developing and evaluating this method was based on the fact that the author’s team has been working on this issue (improvement of accuracy measurement of radio altimeter) for over 20 years. Some flight tests and later also exterior measurements were carried out earlier. As real flight tests are costly and therefore not feasible in conventional conditions, the authors tested the simulation of dynamic altitude change in several ways, by real measurements on aircraft and subsequently by measurements outdoors—in outside conditions. The determining of methodological error from this measurement is shown on Figure 13.

The measuring and recording apparatus, together with the radio altimeter and the bracket for mounting the antennas, were installed on the car, which was moved towards and backwards to the reflecting panel. This method required extensive preparations and depended on external meteorological conditions and a clutter-free environment. That led to the need to move experimental measurements from the exterior to the interior. In the interior (in buildings and hangars), there was an insurmountable problem—unwanted/parasitic reflections from the surroundings (walls, objects, the earth’s surface), which overwhelm the evaluation circuits of the radio altimeter so that it is unable to measure the radio altitude. To suppress these unwanted reflections, the authors designed a special reflection panel. This reflection panel with the measurement method was successfully patented after extensive testing.

As part of testing the new method, we performed a series of indoor and outdoor measurements. As part of the evaluation (flight measurements, outside measurements, and laboratory measurements), we concluded that the course of the methodological error has a constant value in the entire measured range (from 0 m to 750 m). This means, that the value of methodological error is the same at any measured radio altitude, so it is not necessary to perform measurement in whole radio altimeter measurement range, i.e., from 0 m to 750 m. In terms of usage of radio altimeters, we are focusing on its measurement accuracy mostly in small altitudes. As stated in the introduction, radio altitude information is crucial for the entire group of aviation systems. If we choose the most critical function, it is information about the actual altitude above the runway for the automatic landing system and the altitude above terrain for anti-collision systems. At that exact moment, the height is measured in the mentioned range of 0–20 m (decision height), which is crucial for the pilot. This is also why radar altimeter indicators are scaled nonlinear; they are more precise in the first 50 m range, usually with the measurement step by 0.3–1 m (1–3.2 ft). In the case of flight at a higher altitude, another type of flight altitude measurement is used, like a barometric altimeter or GPS altitude, which works on a different principle, and is used as a matter of priority. This is the main reason why all measurements were done in the range from 0 m to 30 m.

Simulating a dynamic change in height is also possible using a dedicated and commercially available test apparatus, such as the Aeroflex ALT 8000. This device can be connected to an existing onboard radio altimeter installation and measure receiver sensitivity and parameter stability (carrier frequency, modulation frequency) and verify exact values of indicated height for onboard systems such as autoland, flare, decision height, etc. However, the purchase price of such a device is relatively high. Furthermore, it does not consider all the effects mentioned above on the accuracy of radio height measurement and does not allow the determination of the exact value of the methodological error. In addition, it connects to the existing installation on the aircraft, which requires operator access directly to the aircraft, which we find to be a disadvantage of this method.

## 4. Conclusions

Radio altimeters are used for precise measurement of the clearance height of aircraft over terrain or obstacles. The typical accuracy of this measurement is from ±0.30 m to ±0.75 m, and the parameter with the most influence over the accuracy of height measurement is the methodological error. Manufacturers usually provide the value of this error, and it is no longer examined over the lifetime of any radio altimeter. However, the practice shows that in some scenarios, this value can change in time and affects height measurement accuracy. As the radio altimeter is the only onboard sensor which provides crucial information on exact clearance height for entire crucial aircraft systems, precise measurement is mandatory.

To clarify that radio altimeter parameters are in corresponding tolerances, the authors proposed a new method for determining the value of the methodological error and its effect on the resulting error of measurement of the radio altitude. The proposed method is laboratory-based and can simulate conditions like in operation on real aircraft. In terms of qualitative assessment, this method, as it simulates the operation of the radar altimeter together with most possible inside and outside factors, can detect the generation of random and systemic interfering signals that may have a negative effect on the accuracy of height measurement. The output of measurement—the numerical value of the methodological error—can be imagined as something like a “snapshot” of the current device and statistically examined in time if parameters are degraded.

The radio altimeter methodological error, which is directly related to the height measurement accuracy, is usually determined theoretically, as it is based on its basic electrical parameters. Subsequently, it is assumed that the radio altimeter has its accuracy determined this way throughout its technical life. The presented method can evaluate the technical condition of the radio altimeter in terms of height measurement accuracy at any stage of its technical life. This has not yet been possible in the aviation industry (with an aircraft operator). The presented method is simple and can be implemented in any laboratory environment without high additional costs. It could stimulate the interest of aircraft operators in using the study to evaluate the impact of long-term operation of radio altimeters on the accuracy of altitude measurements. The results of measurements presented in this work suggest that the implementation of such a study would be possible in practice. With the help of graphical evaluation, it is possible to register any inaccuracy in the setting of its parameters or imperfections in the operation of any circuit of a radio altimeter. Suppose the radio altimeter shows even a slight discrepancy with the required parameters. In that case, the course of the increase of the difference frequency will not be linear, and in terms of methodological error, it would not be symmetric. The quality and accuracy of recording the methodological error of the radio altimeter Δ*H* depend, among other things, on the height sensing speed, i.e., on the speed of movement of the antennas during the measurement.

By adding other elements of the measuring chain, this method can also be suitable for testing actual problems of radio altitude measurements affected by new technologies, i.e., 5G interference.

## Figures and Tables

**Table 1:** Percentual factors of radio altitude measurement errors.

Part of Overall Measurement Error	The Percentage Value of Overall Error	The Numerical Value of Overall Error
Methodological error	70%	±0.525 m
FM parameter instability error	20%	±0.150 m
Radio altimeter dynamic error	6%	±0.045 m
Remaining measurement errors	4%	±0.030 m

**Table 2:** Control measurements of methodological error of RV5 radio altimeter.

Measurement	ΔH of Unit 1	ΔH of Unit 2	ΔH of Unit 3	ΔH of Unit 4
1	±75.00 cm	±74.06 cm	±75.00 cm	±75.94 cm
2	±75.00 cm	±74.06 cm	±75.00 cm	±75.00 cm
3	±75.94 cm	±75.00 cm	±75.00 cm	±75.94 cm
4	±75.94 cm	±75.00 cm	±75.94 cm	±75.94 cm
5	±75.94 cm	±75.94 cm	±75.94 cm	±75.00 cm
